# Ethyl 4-(4-chloro­phen­yl)-6-methyl-2-thioxo-1,2,3,4-tetra­hydro­pyrimidine-5-carboxyl­ate

**DOI:** 10.1107/S1600536809037453

**Published:** 2009-09-26

**Authors:** Susanta K. Nayak, K. N. Venugopala, Deepak Chopra, Thavendran Govender, Hendrik G. Kruger, Glenn E. M. Maguire, T. N. Guru Row

**Affiliations:** a Solid State and Structural Chemistry Unit, Indian Institute of Science, Bangalore 560 012, India; b School of Chemistry, University of KwaZulu-Natal, Durban 4000, South Africa; c Department of Chemistry, Indian Institute of Science Education and Research, Bhopal 462 023, India; d School of Pharmacy and Pharmacology, University of Kwazulu-Natal, Durban 4000, South Africa

## Abstract

In the title compound, C_14_H_15_ClN_2_O_2_S, the tetra­hydro­pyrimidine ring adopts a twisted boat conformation with the carbonyl group in an *s-trans* conformation with respect to the C=C double bond of the six-membered tetra­hydro­pyrimidine ring. The mol­ecular conformation is determined by an intra­molecular C—H⋯π inter­action. The crystal structure is further stabilized by inter­molecular N—H⋯O mol­ecular chains and centrosymmetric N—H⋯S dimers.

## Related literature

For background to the applications of poly-functionalized dihydro­pyrimidines, see: Corey & Cheng (1995 ▶); Hurst & Hull (1961 ▶); Jauk *et al.* (2000 ▶); Kappe (2000 ▶); Mayer *et al.* (1999 ▶). For ring puckering parameters, see: Cremer & Pople (1975 ▶).
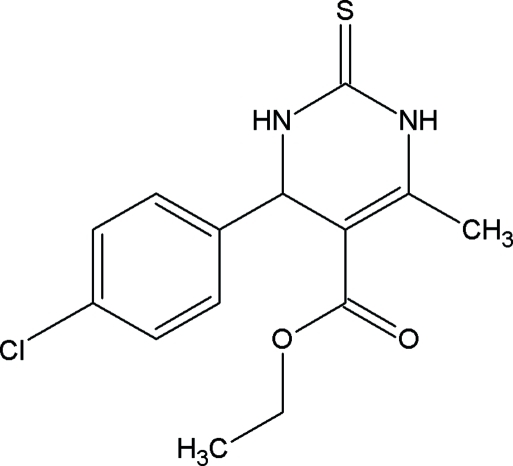


## Experimental

### 

#### Crystal data

C_14_H_15_ClN_2_O_2_S
*M* *_r_* = 310.80
Triclinic, 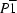

*a* = 7.3420 (3) Å
*b* = 9.4895 (4) Å
*c* = 12.0425 (5) Å
α = 73.823 (4)°
β = 88.512 (3)°
γ = 70.264 (4)°
*V* = 756.32 (6) Å^3^
*Z* = 2
Mo *K*α radiation
μ = 0.39 mm^−1^
*T* = 292 K
0.24 × 0.22 × 0.18 mm

#### Data collection

Oxford Diffraction Xcalibur diffractometer with Eos (Nova) detector
Absorption correction: multi-scan (*CrysAlis Pro*; Oxford Diffraction, 2009 ▶) *T*_min_ = 0.902, *T*_max_ = 0.933
16944 measured reflections
2960 independent reflections
2232 reflections with *I* > 2σ(*I*)
*R*_int_ = 0.040

#### Refinement

*R*[*F* ^2^ > 2σ(*F* ^2^)] = 0.053
*wR*(*F* ^2^) = 0.161
*S* = 1.09
2960 reflections
183 parameters
H-atom parameters constrained
Δρ_max_ = 0.48 e Å^−3^
Δρ_min_ = −0.37 e Å^−3^

### 

Data collection: *CrysAlis Pro* (Oxford Diffraction, 2009 ▶); cell refinement: *CrysAlis Pro*; data reduction:  *CrysAlis Pro*; program(s) used to solve structure: *SHELXL97* (Sheldrick, 2008 ▶); program(s) used to refine structure: *SHELXL97* (Sheldrick, 2008 ▶); molecular graphics: *ORTEP-3 for Windows* (Farrugia, 1997 ▶) and *CAMERON* (Watkin *et al.*, 1993 ▶); software used to prepare material for publication: *PLATON* (Spek, 2009 ▶).

## Supplementary Material

Crystal structure: contains datablocks global, I. DOI: 10.1107/S1600536809037453/sj2653sup1.cif

Structure factors: contains datablocks I. DOI: 10.1107/S1600536809037453/sj2653Isup2.hkl

Additional supplementary materials:  crystallographic information; 3D view; checkCIF report

## Figures and Tables

**Table 1:** Hydrogen-bond geometry (Å, °)

*D*—H⋯*A*	*D*—H	H⋯*A*	*D*⋯*A*	*D*—H⋯*A*
N1—H1⋯O1^i^	0.86	2.25	3.077 (3)	161
N2—H2⋯S1^ii^	0.86	2.49	3.323 (3)	164
C14—H14⋯*Cg*1	0.93	2.67	3.146 (4)	113

Symmetry codes: (i) 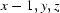; (ii) 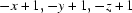. *Cg*1 is the centroid of the C2=C3 double bond.

**Fig. 1.**
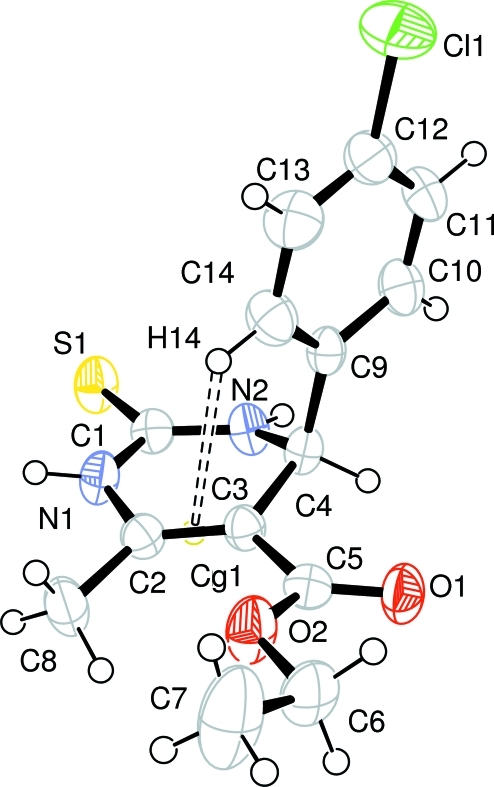
: The structure of the title compound showing the atom labelling Scheme with displacement ellipsoids for non-H atoms at the 50% probability level. The dotted line shows the C—H···π intramolecular interactions. Cg1 (the orange open circle) denotes the center of gravity of the C2=C3 bond.

**Fig. 2.**
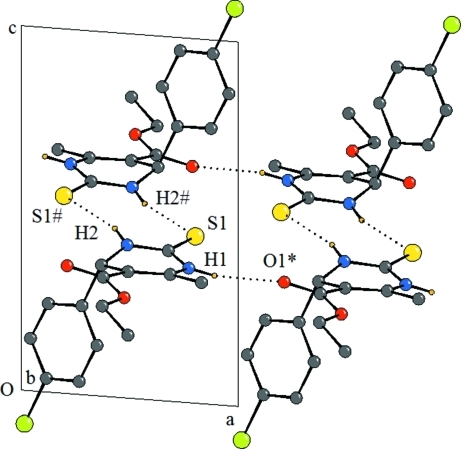
: The crystal packing showing the molecular chains of N—H···O hydrogen bonds and N—H···S centrosymmetric dimers. Molecules at # and * have the symmetry codes (- x + 1, - y + 1, - z + 1) and (x - 1, y, z) respectively.

## supplementary crystallographic information

### Comment

The logic of chemical reactivity (Corey & Cheng, 1995) has found application in the rational design of a variety of drug molecules. One such class of compounds is the "Bignelli compounds". These are poly-functionalized dihydropyrimidine (DHPM's) exhibiting a broad range of therapeutic and pharmacological properties (Kappe, 2000) namely, antiviral (Hurst *et al.*, 1961), antimimotic (Mayer *et al.*,1999) and calcium channel modulators (Jauk *et al.*, 2000). In view of immense range of applications of this class of compounds we have undertaken a single-crystal determination of the title compound.

The tetrahydropyrimidine ring adopts a twist boat conformation. The puckering parameters (Cremer & Pople 1975) are Q = 0.277 (3) Å, θ(2) = 108.1 (3)° and φ(2) = 349.1 (6)° respectively. The orientation of the chloro-phenyl moiety is such that it bisects the twist boat conformation of the tetrahydropyrimidine ring, the C9—C4—C3—C5 torsion angle being 77.4 (3)°. The molecular conformation is stabilized by an intramolecular C—H···π interaction (2.67 Å, 113°) wherein the aryl hydrogen H14 is oriented towards the π electrons of the C2=C3 double bond (Figure 1). The crystal structure is further stabilized by centrosymmetric N—H···S dimers and N—H···O hydrogen bonds forming molecular chains along the crystallographic *a* axis (Figure 2).

### Experimental

A mixture of ethylacetoacetate (0.1 mol), *para* chlorosubstituted benzaldehyde (0.1 mol) and thiourea was refluxed in 50.0 mL of ethanol for 2.0 hrs in presence of concentrated hydrochloric acid as catalyst. The reaction completion was monitored through thin layer chromatography and and, on completion, the products were poured into ice cold water. The precipitate obtained was filtered, dried and crystallized from methanol to obtain the title compound.

### Refinement

All H atoms were positioned geometrically, C—H = 0.93 Å, 0.96 Å, 0.97 Å, 0.98Å for aromatic, methyl, methylene and methine hydrogen respectively and N—H = 0.86 Å and all refined using a riding model with *U*_iso_(H)= 1.2 *U*_eq_(C, N) for aromatic and amine hydrogen and 1.5 *U*_eq_(C) for methyl, methylene and methine H atoms respectively.

### Crystal data

**Table:**

C_14_H_15_ClN_2_O_2_S	*Z* = 2
*M**_r_* = 310.80	*F*(000) = 324
Triclinic, *P*1	*D*_x_ = 1.365 Mg m^−^^3^
Hall symbol: -P 1	Mo *K*α radiation, λ = 0.7107 Å
*a* = 7.3420 (3) Å	Cell parameters from 340 reflections
*b* = 9.4895 (4) Å	θ = 1.0–28.0°
*c* = 12.0425 (5) Å	µ = 0.39 mm^−^^1^
α = 73.823 (4)°	*T* = 292 K
β = 88.512 (3)°	Block, colorless
γ = 70.264 (4)°	0.24 × 0.22 × 0.18 mm
*V* = 756.32 (6) Å^3^	

### Data collection

**Table:**

Oxford Diffraction Xcalibur with Eos (Nova) detector diffractometer	2960 independent reflections
Radiation source: Enhance (Mo) X-ray Source	2232 reflections with *I* > 2σ(*I*)
graphite	*R*_int_ = 0.040
Detector resolution: 16.0839 pixels mm^-1^	θ_max_ = 26.0°, θ_min_ = 3.3°
ω scans	*h* = −9→9
Absorption correction: multi-scan (*CrysAlis PRO*; Oxford Diffraction, 2009)	*k* = −11→11
*T*_min_ = 0.902, *T*_max_ = 0.933	*l* = −14→14
16944 measured reflections	

### Refinement

**Table:**

Refinement on *F*^2^	Primary atom site location: structure-invariant direct methods
Least-squares matrix: full	Secondary atom site location: difference Fourier map
*R*[*F*^2^ > 2σ(*F*^2^)] = 0.053	Hydrogen site location: inferred from neighbouring sites
*wR*(*F*^2^) = 0.161	H-atom parameters constrained
*S* = 1.09	*w* = 1/[σ^2^(*F*_o_^2^) + (0.094*P*)^2^ + 0.1394*P*] where *P* = (*F*_o_^2^ + 2*F*_c_^2^)/3
2960 reflections	(Δ/σ)_max_ < 0.001
183 parameters	Δρ_max_ = 0.48 e Å^−^^3^
0 restraints	Δρ_min_ = −0.37 e Å^−^^3^

### Special details

**Table:**

Geometry. All e.s.d.'s (except the e.s.d. in the dihedral angle between two l.s. planes) are estimated using the full covariance matrix. The cell e.s.d.'s are taken into account individually in the estimation of e.s.d.'s in distances, angles and torsion angles; correlations between e.s.d.'s in cell parameters are only used when they are defined by crystal symmetry. An approximate (isotropic) treatment of cell e.s.d.'s is used for estimating e.s.d.'s involving l.s. planes.
Refinement. Refinement of *F*^2^ against ALL reflections. The weighted *R*-factor *wR* and goodness of fit *S* are based on *F*^2^, conventional *R*-factors *R* are based on *F*, with *F* set to zero for negative *F*^2^. The threshold expression of *F*^2^ > σ(*F*^2^) is used only for calculating *R*-factors(gt) *etc*. and is not relevant to the choice of reflections for refinement. *R*-factors based on *F*^2^ are statistically about twice as large as those based on *F*, and *R*- factors based on ALL data will be even larger.

### Fractional atomic coordinates and isotropic or equivalent isotropic displacement parameters (Å^2^)

**Table:**

	*x*	*y*	*z*	*U*_iso_*/*U*_eq_	
S1	0.19746 (10)	0.59669 (9)	0.54217 (7)	0.0474 (3)	
Cl1	0.98420 (16)	0.22640 (13)	1.09156 (8)	0.0841 (4)	
N2	0.5115 (3)	0.6617 (3)	0.57987 (18)	0.0368 (5)	
H2	0.5687	0.6038	0.5368	0.044*	
C3	0.5079 (4)	0.8759 (3)	0.6529 (2)	0.0337 (6)	
N1	0.2287 (3)	0.8143 (3)	0.6307 (2)	0.0395 (5)	
H1	0.1099	0.8288	0.6463	0.047*	
C4	0.6301 (4)	0.7183 (3)	0.6403 (2)	0.0343 (6)	
H4	0.7360	0.7320	0.5922	0.041*	
O2	0.5259 (3)	1.0762 (3)	0.7252 (2)	0.0562 (6)	
O1	0.7882 (3)	0.9466 (2)	0.64949 (19)	0.0501 (5)	
C1	0.3222 (4)	0.6937 (3)	0.5869 (2)	0.0352 (6)	
C9	0.7198 (3)	0.5977 (3)	0.7561 (2)	0.0330 (6)	
C2	0.3137 (4)	0.9158 (3)	0.6519 (2)	0.0352 (6)	
C5	0.6218 (4)	0.9691 (3)	0.6732 (2)	0.0373 (6)	
C14	0.6506 (4)	0.6140 (4)	0.8616 (2)	0.0467 (7)	
H14	0.5469	0.7027	0.8633	0.056*	
C6	0.6261 (5)	1.1724 (4)	0.7526 (3)	0.0571 (8)	
H6A	0.7528	1.1073	0.7916	0.069*	
H6B	0.6434	1.2452	0.6821	0.069*	
C8	0.1693 (4)	1.0610 (4)	0.6676 (3)	0.0520 (8)	
H8A	0.2170	1.1461	0.6395	0.078*	
H8B	0.1488	1.0468	0.7485	0.078*	
H8C	0.0490	1.0840	0.6251	0.078*	
C10	0.8750 (4)	0.4642 (4)	0.7572 (3)	0.0472 (7)	
H10	0.9247	0.4523	0.6873	0.057*	
C12	0.8839 (4)	0.3695 (4)	0.9625 (3)	0.0499 (7)	
C11	0.9569 (4)	0.3507 (4)	0.8560 (3)	0.0489 (7)	
H11	1.0597	0.2617	0.8539	0.059*	
C13	0.7329 (5)	0.5008 (4)	0.9644 (3)	0.0532 (8)	
H13	0.6856	0.5140	1.0345	0.064*	
C7	0.5083 (6)	1.2561 (6)	0.8271 (5)	0.1004 (17)	
H7A	0.5077	1.1839	0.9009	0.151*	
H7B	0.3781	1.3084	0.7922	0.151*	
H7C	0.5608	1.3317	0.8379	0.151*	

### Atomic displacement parameters (Å^2^)

**Table:**

	*U*^11^	*U*^22^	*U*^33^	*U*^12^	*U*^13^	*U*^23^
S1	0.0396 (4)	0.0547 (5)	0.0629 (5)	−0.0215 (3)	0.0076 (3)	−0.0340 (4)
Cl1	0.0859 (7)	0.0806 (7)	0.0595 (6)	−0.0202 (6)	−0.0165 (5)	0.0120 (5)
N2	0.0311 (12)	0.0444 (13)	0.0402 (11)	−0.0104 (10)	0.0019 (9)	−0.0236 (10)
C3	0.0304 (13)	0.0358 (14)	0.0366 (13)	−0.0117 (11)	0.0014 (10)	−0.0124 (11)
N1	0.0330 (12)	0.0468 (13)	0.0508 (13)	−0.0190 (10)	0.0131 (10)	−0.0271 (11)
C4	0.0300 (13)	0.0409 (14)	0.0386 (13)	−0.0156 (11)	0.0079 (10)	−0.0179 (11)
O2	0.0448 (12)	0.0589 (14)	0.0878 (16)	−0.0276 (10)	0.0155 (11)	−0.0453 (13)
O1	0.0322 (11)	0.0551 (13)	0.0722 (14)	−0.0202 (9)	0.0073 (9)	−0.0265 (11)
C1	0.0369 (14)	0.0388 (14)	0.0316 (12)	−0.0127 (11)	0.0040 (10)	−0.0134 (11)
C9	0.0277 (13)	0.0393 (14)	0.0378 (13)	−0.0141 (11)	0.0041 (10)	−0.0171 (11)
C2	0.0341 (14)	0.0353 (14)	0.0376 (13)	−0.0123 (11)	0.0015 (10)	−0.0121 (11)
C5	0.0404 (16)	0.0338 (14)	0.0371 (13)	−0.0120 (12)	0.0017 (11)	−0.0098 (11)
C14	0.0449 (17)	0.0473 (16)	0.0463 (16)	−0.0090 (13)	0.0060 (13)	−0.0195 (14)
C6	0.056 (2)	0.0570 (19)	0.079 (2)	−0.0327 (16)	0.0092 (17)	−0.0356 (18)
C8	0.0314 (15)	0.0469 (17)	0.082 (2)	−0.0096 (13)	0.0046 (14)	−0.0304 (17)
C10	0.0379 (15)	0.0524 (18)	0.0508 (17)	−0.0091 (13)	0.0088 (13)	−0.0224 (15)
C12	0.0429 (16)	0.0570 (18)	0.0457 (16)	−0.0185 (14)	−0.0079 (13)	−0.0056 (14)
C11	0.0324 (15)	0.0459 (17)	0.0593 (18)	−0.0023 (13)	0.0021 (13)	−0.0147 (15)
C13	0.060 (2)	0.062 (2)	0.0397 (16)	−0.0214 (16)	0.0062 (14)	−0.0179 (15)
C7	0.079 (3)	0.130 (4)	0.152 (4)	−0.063 (3)	0.045 (3)	−0.103 (4)

### Geometric parameters (Å, °)

**Table:**

S1—C1	1.688 (3)	C2—C8	1.486 (4)
Cl1—C12	1.735 (3)	C14—C13	1.382 (4)
N2—C1	1.324 (3)	C14—H14	0.9300
N2—C4	1.464 (3)	C6—C7	1.441 (5)
N2—H2	0.8600	C6—H6A	0.9700
C3—C2	1.345 (3)	C6—H6B	0.9700
C3—C5	1.474 (4)	C8—H8A	0.9600
C3—C4	1.510 (3)	C8—H8B	0.9600
N1—C1	1.359 (3)	C8—H8C	0.9600
N1—C2	1.390 (3)	C10—C11	1.349 (4)
N1—H1	0.8600	C10—H10	0.9300
C4—C9	1.528 (4)	C12—C13	1.365 (5)
C4—H4	0.9800	C12—C11	1.411 (4)
O2—C5	1.330 (3)	C11—H11	0.9300
O2—C6	1.455 (3)	C13—H13	0.9300
O1—C5	1.208 (3)	C7—H7A	0.9600
C9—C14	1.386 (4)	C7—H7B	0.9600
C9—C10	1.386 (4)	C7—H7C	0.9600
			
C1—N2—C4	124.5 (2)	C7—C6—O2	107.4 (3)
C1—N2—H2	117.7	C7—C6—H6A	110.2
C4—N2—H2	117.7	O2—C6—H6A	110.2
C2—C3—C5	126.0 (2)	C7—C6—H6B	110.2
C2—C3—C4	119.9 (2)	O2—C6—H6B	110.2
C5—C3—C4	113.9 (2)	H6A—C6—H6B	108.5
C1—N1—C2	123.8 (2)	C2—C8—H8A	109.5
C1—N1—H1	118.1	C2—C8—H8B	109.5
C2—N1—H1	118.1	H8A—C8—H8B	109.5
N2—C4—C3	109.1 (2)	C2—C8—H8C	109.5
N2—C4—C9	110.3 (2)	H8A—C8—H8C	109.5
C3—C4—C9	113.21 (19)	H8B—C8—H8C	109.5
N2—C4—H4	108.0	C11—C10—C9	122.5 (3)
C3—C4—H4	108.0	C11—C10—H10	118.7
C9—C4—H4	108.0	C9—C10—H10	118.7
C5—O2—C6	118.1 (2)	C13—C12—C11	120.0 (3)
N2—C1—N1	116.0 (2)	C13—C12—Cl1	119.6 (2)
N2—C1—S1	123.59 (19)	C11—C12—Cl1	120.5 (2)
N1—C1—S1	120.36 (19)	C10—C11—C12	118.9 (3)
C14—C9—C10	117.7 (3)	C10—C11—H11	120.6
C14—C9—C4	122.9 (2)	C12—C11—H11	120.6
C10—C9—C4	119.5 (2)	C12—C13—C14	119.8 (3)
C3—C2—N1	118.7 (2)	C12—C13—H13	120.1
C3—C2—C8	128.3 (2)	C14—C13—H13	120.1
N1—C2—C8	112.9 (2)	C6—C7—H7A	109.5
O1—C5—O2	123.2 (2)	C6—C7—H7B	109.5
O1—C5—C3	123.5 (2)	H7A—C7—H7B	109.5
O2—C5—C3	113.2 (2)	C6—C7—H7C	109.5
C13—C14—C9	121.2 (3)	H7A—C7—H7C	109.5
C13—C14—H14	119.4	H7B—C7—H7C	109.5
C9—C14—H14	119.4		
			
C1—N2—C4—C3	−31.3 (3)	C1—N1—C2—C8	163.8 (3)
C1—N2—C4—C9	93.6 (3)	C6—O2—C5—O1	1.1 (4)
C2—C3—C4—N2	24.4 (3)	C6—O2—C5—C3	178.3 (2)
C5—C3—C4—N2	−159.3 (2)	C2—C3—C5—O1	−163.6 (3)
C2—C3—C4—C9	−98.8 (3)	C4—C3—C5—O1	20.4 (4)
C5—C3—C4—C9	77.4 (3)	C2—C3—C5—O2	19.2 (4)
C4—N2—C1—N1	15.8 (4)	C4—C3—C5—O2	−156.7 (2)
C4—N2—C1—S1	−165.7 (2)	C10—C9—C14—C13	−0.3 (4)
C2—N1—C1—N2	9.3 (4)	C4—C9—C14—C13	178.6 (3)
C2—N1—C1—S1	−169.2 (2)	C5—O2—C6—C7	−169.1 (3)
N2—C4—C9—C14	−103.7 (3)	C14—C9—C10—C11	1.1 (4)
C3—C4—C9—C14	18.9 (3)	C4—C9—C10—C11	−177.8 (3)
N2—C4—C9—C10	75.2 (3)	C9—C10—C11—C12	−1.0 (5)
C3—C4—C9—C10	−162.3 (2)	C13—C12—C11—C10	0.0 (5)
C5—C3—C2—N1	179.7 (2)	Cl1—C12—C11—C10	179.8 (2)
C4—C3—C2—N1	−4.6 (4)	C11—C12—C13—C14	0.8 (5)
C5—C3—C2—C8	1.6 (5)	Cl1—C12—C13—C14	−179.0 (2)
C4—C3—C2—C8	177.3 (3)	C9—C14—C13—C12	−0.7 (5)
C1—N1—C2—C3	−14.6 (4)		

### Hydrogen-bond geometry (Å, °)

**Table:**

*D*—H···*A*	*D*—H	H···*A*	*D*···*A*	*D*—H···*A*
N1—H1···O1^i^	0.86	2.25	3.077 (3)	161
N2—H2···S1^ii^	0.86	2.49	3.323 (3)	164
C14—H14···Cg1	0.93	2.67	3.146 (4)	113

Symmetry codes: (i) *x*−1, *y*, *z*; (ii) −*x*+1, −*y*+1, −*z*+1.
